# Comparison of surgical and audiological outcomes of endoscopic and microscopic approach in stapes surgery

**DOI:** 10.12669/pjms.35.5.439

**Published:** 2019

**Authors:** Secaattin Gulsen, Erkan Karatas

**Affiliations:** 1 Dr. Secaattin Gulsen, MD. ENT Specialist Department of Otorhinolaryngology, Dr. Ersin Arslan Training and Research Hospital, Gaziantep, Turkey; 2 Prof. Dr. Erkan Karatas, MD. Department of Otorhinolaryngology, Inonu University Faculty of Medicine, Malatya, Turkey

**Keywords:** Stapes surgery, Stapedotomy, Endoscope, Otosclerosis

## Abstract

**Objective:**

The main objectives of the present study were to compare the surgical and audiological outcomes of endoscopic and microscopic approach in stapes surgery.

**Methods:**

Sixty-one patients who underwent the stapes surgery with the endoscopic and microscopic approach between January 2012-November 2018 were included in the study. Patients were divided into two groups as a Group-I (endoscopic) and Group-II (microscopic). The audiometric measurements, duration of surgery, intraoperative findings and complications were recorded and evaluated retrospectively.

**Results:**

Mean operative time for the Group-I and II was 45.1±8.4 minutes and 48.7±5.6 minutes, respectively (p>0.05). The preoperative and postoperative average air-bone gap in the Group-I was 27.8±7.2 dB and 8.7±3.4 dB and these values in Group-II were 30.2±5.1 dB and 7.4±4.8 dB, respectively (p<0.001). The requirement of chorda tympani nerve manipulation and scutum curettage were significantly less in Group-I as compared Group-II (p<0.05). Dysgeusia and postoperative pain were observed significantly higher ratios in Group-II relative to Group-I (p<0.05). There was no significant difference between endoscopic and microscopic approach in stapes surgery in terms of difficulty of prosthesis insertion (p>0.05).

**Conclusion:**

Endoscopic stapes surgery provides comparable audiological outcomes, shorter operative times, fewer complications rates, and more minimally invasive surgery, relative to the microscopic approach.

## INTRODUCTION

Otosclerosis, which is characterized by bone resorption and sclerotic bone formation in temporal bone and might be resulting in either conductive or mixed type hearing loss, was first described by Valsalva.^1^ In stapes surgery (SS); different surgical techniques, approaches and prostheses in order to restore sound transmission have been described. Stapedectomy was first described by Shea.^2^

Currently, a small fenestration stapedotomy proposed by Ugo Fish is a widely accepted procedure for otosclerosis surgery.^3^ Poe has first described the laser-assisted endoscopic stapes surgery (ESS) in 2000, soon after Tarabichi reported preliminary results of ESS in 1999.^4^,^5^ Recently endoscopes as a primary or auxiliary intensively started to be used in SS.^6^-^8^ The endoscopes provide excellent and panoramic visualization of the complex middle ear anatomy and particularly stapedial structures.^9^ Nonetheless, microscopic transcanal or endaural approach is the most preferred technique in SS currently. Although microscopes provide good magnification and let both hands use, sufficient exposure of stapedial structures might not be possible under microscopic approach without bone curettage.^10^-^13^

In the present study, authors comprehensively compared the surgical and functional outcomes of endoscopic and microscopic approach in SS.

## METHODS

The present study included 61 consecutive patients who underwent endoscopic and microscopic SS between January 2012-November 2018 at Dr. Ersin Arslan Training and Research Hospital and Private Hatem Hospital. The informed consent and local ethical committee approval were obtained prior to the study conduction. Pure tone audiometry (PTA), tympanogram, and stapes reflex threshold was performed preoperatively. The conductive type of hearing loss with stapes fixation was the inclusion criteria of the patients to the study. Revision cases were not included in the study. All patients who underwent endoscopic SS was operated by the first author. In the microscopic group, of the 20 of 29 patients were operated by the first author and the remaining nine patients were operated by the other surgeon. The temporal bone computed tomography was obtained from all patients to assure whether that they have any other condition causing a conductive type of hearing loss such as tympanosclerosis, ossicular dislocation or superior semi-circular canal dehiscence syndrome. Patients were divided into two groups as I and II respecting the endoscopic or microscopic approach preferred, respectively. Pre-operative and post-operative PTA, intraoperative findings, complications, and operative time were retrospectively analyzed. Air-conduction thresholds (ACT) and bone-conduction thresholds (BCT) at the frequencies of 0.5, 1, 2, and 4 kHz were measured preoperatively, and six months after surgery. The preoperative and postoperative air-bone gap (ABG) values calculated. PTA results and ACT, BCT and ABG values were calculated as suggested by the American Academy of Otolaryngology-Head and Neck Foundation Committee on Hearing and Equilibrium guideline.^12^ Intraoperative findings such as chorda tympani nerve (CTN) manipulation requirement, scutum curettage necessity and the difficulty in insertion of the prosthesis were noted. Requiring more than one attempt for placement of prosthesis was defined as difficulty in the insertion of prosthesis. Patients were inquired for pain, dizziness, and dysgeusia postoperatively. Visual analog scales (VAS) were used to identify the intensity of dizziness and pain postoperatively. The severity of postoperative pain was classified as almost no pain, mild pain non-requiring analgesic or moderate pain requiring analgesic treatment. The intensity of postoperative dizziness was recorded as no dizziness, moderate dizziness with 1st. degree of nystagmus relieved with bed rest and motion restriction, and severe dizziness with 2nd. or 3th. degree nystagmus requiring hospitalization and antivertiginous treatment.

### Surgical Technique

**Fig.1:**
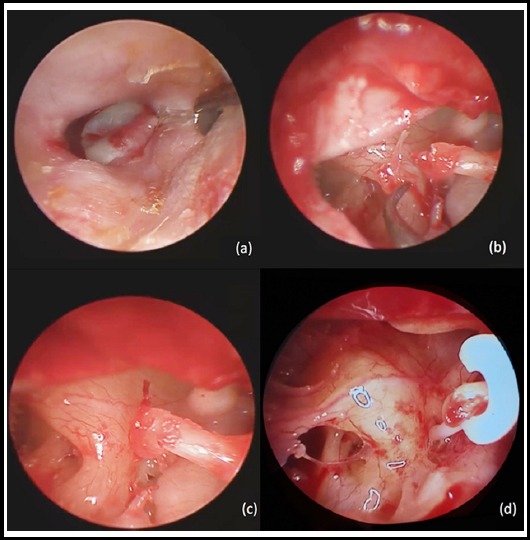
a-d Photographs showing endoscopic stapes surgery steps. Endoscopic transcanal incisions (a). Removal of stapes superstructure (b). Fenestration of footplate (c). An intraoperative view of loop prosthesis inserted (d)

Diluted lidocaine and adrenaline solution (Jetokain®, Adeka, Samsun, Turkey) was applied to four quadrants of external auditory canal (EAC) to diminish bothersome bleedings. After a while, Rosen incision, 1-1.5 cm lateral to the tympanic annulus, was made and tympanomeatal flap elevation performed. Fibrous annulus was separated meticulously from the tympanic sulcus, and middle ear cavity reached. If necessary, curettage of the scutum with preserving CTN was done to provide adequate exposure of stapedial structures, incudostapedial joint and tympanic segment of the facial nerve in Group-I. In microscopic stapes surgery (MSS), an additional endaural incision was performed in required cases whereas the scutum curettage was routinely performed. Stapes fixation and the mobility of the incus and malleus were confirmed by gently palpation of ossicles. A small fenestra on the footplate was created via perforators after separation of the incudostapedial joint, cutting of the stapedial tendon and removal of the stapes superstructure. Then, a measuring rod was utilized for calculating the distance between the long process of incus and footplate. Polytetrafluoroethylene artificial loop prosthesis (Xomed, Jacksonville, Fla, USA), with a length of 4 - 4.5 - 4.75 - 5 mm, with respect to the distance between the long process of incus and footplate with a 0.4 - 0.6 mm shaft thickness, was placed (Fig.1). Tiny Gelfoam® (Ferrosan, Soborg, Denmark) pieces were placed around the fenestra and prosthesis shaft to prevent perilymph fistula and to stabilize prosthesis. The tympanomeatal flap was put back to its’ original position and supported with ciprofloxacin soaked Gelfoam.

### Statistical Analysis

The statistical package for social science (SPSS) 22.0 software was used for descriptive and statistical analysis of the data. Categorical variables between groups were compared using the x^2^ test. Student’s t-test was utilized for analyzing the normally distributed data. Results were presented as mean (±SD), median (range) and n (%). A P value less than 0.05 was considered to be statistically significant.

## RESULTS

In the endoscopic group, there were 15 females (46.9%) and 17 males (53.1%) with the average age of 32.6±10.8 years (range; 19-51). There was 18 (47.4%) left, and 20 (52.6%) right ears in Group-I. The microscopic group included 19 (65.5%) females and 10 (34.5%) males with the average age of 35.6±7.1 years (range; 23-49). Group-II comprised 18 (52.9%) left, and 16 (47.1%) right ears. The transcanal approach (n=38, 100%) was sufficient in the endoscopic group, whereas additional endaural (n=11, 32.3%) incision was required in patients operated via a surgical microscope in order to obtain adequate exposure. Average postoperative follow-up duration was 13.2 (range 8-18) and 12.1 (range 9-15) months in Group-I and Group-II, respectively.

Postoperative ABG and ACT values were improved significantly in both groups as presented in Table-I (p<0.001). However, there was no statistically significant difference between groups regarding auditory gain (p > 0.05). The mean operative time was 45.1±8.4 min (range 36-51 min) for the Group-I, whereas it was 48.7±5.6 min (range 39-53 min) for the Group-II (p>0.05).

**Table I:** Comparison of audiologic results of the Group I and II.

PTA Results	Group I		Group II		*P value

	Preoperative (mean-SD)	Postoperative (mean-SD)	Preoperative (mean-SD)	Postoperative (mean-SD)	
ACT (dB)	38.7 ± 6.1	18.4± 2.9	40.1± 5.7	16.8± 4.2	<0.001
BCT (dB)	9.6± 2.3	10.8± 2.5	10.2± 3.1	9.4± 2.7	>0.05
ABG (dB)	27.8 ± 7.2	8.7 ± 3.4	30.2 ± 5.1	7.4 ± 4.8	<0.001
Auditory gain (dB)	22.8± 7.1		19.1± 5.8		>0.05

* A P value <0.05 was considered to be statistically significant.

Scutum curettage was required in 26 (68.4%) cases in Group-I in order to achieve appropriate exposure. However, resection of scutum was routinely performed in Group-II (n=34, 100%). Scutum curettage necessity was significantly lower in Group-I as compared to Group-II (p<0.05). CTN manipulation was required in 13 (34.2%) and 22 (64.7%) cases in Group-I and II, respectively (p<0.05). Six (15.7%) patients in Group-I and 4 (11.7%) patients in Group-II was required more than one attempt for placement of the prosthesis. There was no significant difference between endoscopic and microscopic approach regarding the difficulty of prosthesis insertion (p>0.05).

Intraoperative and postoperative complications are presented in Table-II. The number of cases suffering from pain and dysgeusia postoperatively was significantly high in Group-II relative to Group-I (Table-II). There was no significant difference between groups regarding postoperative dizziness (p>0.05). Iatrogenic minimal tympanic membrane perforation (n=1) and floating footplate (n=1) have occurred in patients who underwent ESS. In the microscopic group, numbness at the auricle (n=2) owing to endaural incision and inadvertent CTN laceration (n=1) due to the excessive curettage of the scutum has occurred (Table-II). Sensorineural hearing loss (SNHL), permanent or temporary facial nerve dysfunction, and dizziness not observed in any of patients in both groups postoperatively.

**Table II:** Comparison of complication rates between groups.

Complications	Group I	Group II	*P value
Pain			
Almost no pain	22 (57.9%)	10 (29.5%)	<0.05
Mild pain non-requiring analgesic	10 (26.3%)	6 (17.6%)	<0.05
Moderate pain requiring analgesic	6 (15.8%)	18 (52.9%)	<0.001
Dizziness			
Almost no dizziness	31 (81.6%)	28 (82.3%)	>0.05
Moderate dizziness	4 (10.5%)	4 (11.8%)	>0.05
Severe dizziness	3 (7.9%)	2 (5.9%)	>0.05
Dysgeusia	7 (18.4%)	15 (44.1%)	<0.05
Iatrogenic perforation	1 (2.6%)	0	
Floating footplate	1 (2.6%)	0	
Numbness at auricle	0	2 (5.8%)	
CTN laceration	0	1 (2.9%)	

* A P value less than 0.05 was considered to be statistically significant.

## DISCUSSION

In order to view concealed areas at the middle ear space while working with the surgical microscopes, extensive curettage of scutum and canaloplasty which results in prolonged operative times and certain complications such as CTN injury, postoperative pain, and ossicular dislocations may be required.^6^-^11^ In this context, endoscopes provide considerable benefits such as improved visualization, panoramic view of the complex middle ear structures, and ease in exploring the concealed areas by simply pushing forward and rotating it around.^7^,^13^ Furthermore, according to our opinion, ESS might be better for training and educational purposes since the endoscopes facilitate the understanding of the surgical procedure and provide a detailed and a wide-angle view of ossicles, horizontal segment of the facial nerve, round window and particularly stapedial structures. On the other hand, one-handed surgery, prolonged learning curve, and lack of stereoscopic vision are the major limitations of the ESS.^10^,^11^,^13^ To eliminate those limitations, attaching the endoscope to a holder in order to use both hands, and the use of 3D endoscopes to provide stereoscopic vision was proposed.^14^,^15^ In our opinion, attaching the endoscope to a holder is not a reasonable solution due to the need for frequent cleaning of the endoscope tip and since fixed endoscope may limit the manoeuvres of the surgeon. The 3D endoscopes are being produced wider in diameter and its use might not be convenient in patients with narrow EAC, and they are also expensive systems. Moreover, as the experience in ESS increases gradually, the surgeon becomes accustomed to working with the two-dimensional view thus the lack of stereoscopic vision will be no longer a limiting factor.

Authors argued that the use of conventional endoscopes 4 mm in diameter and 18 cm in length is easier to use and offer a wider angle of view.^16^ In our study, 0 and 30-degree angled, 2.7 mm in diameter and 11 cm in length endoscopes used in ESS and we did not experience any difficulties regarding manipulation ease and visualization. Furthermore, it is predictable to be that classical 4 mm in diameter sinonasal endoscopes may constrict the working space especially in the patients with the narrow EAC and limit the surgeon’s moves.

The average operative time for endoscopic and microscopic approach was ranging between 31.7 - 65.1 minutes and 36.5 - 71.2 minutes, respectively.^6^,^13^,^17^ Unlike other studies, Iannella G. and Magliulo G. reported that mean operative time in microscopic SS was significantly shorter relative to ESS.^11^ In our opinion, despite the prolonged operative times of ESS in the beginning, as the experience increases the duration of surgery might be decreased in a short time of period and become shorter than microscopic approach.

Even though there is no consensus on postoperative audiometric assessment in ESS, success is defined as the regression of postoperative ABG values below to 10, 15 and 20 dB in the literature.^6^,^11^,^13^,^17^ Postoperative ABG value ≤10 dB observed at 28 (87.5%) and 26 (89.7%) patients in the Group-I and Group-II respectively. Postoperative ABG value between 10-20 dB observed at 4 (12.5%) and 3 (10.3%) patients in the Group-I and Group-II, respectively. The present study validated that both approaches are effective in ensuring comparable audiological outcomes in SS (Table-II).

Curettage of scutum is a crucial step in SS for obtaining the adequate exposure of stapes and adjacent structures. This procedure is almost routinely performed in microscopic approaches in order to obtain appropriate exposure of stapedial structures, whereas scutum curettage is required fewer in ESS. Particularly in microscopic approaches, extensive curettage of scutum may result in complications such as CTN injury, impaired taste sensation, subluxation of ossicles, retraction pockets and postoperative higher pain levels.^6^,^8^,^9^ In our study, not only the requirement of scutum curettage but also the amount of scutum resected was less in the Group-I as compared Group-II.

CTN injury and dysgeusia were reported mostly occurred in microscopic approaches. The endoscopic approach provides lower CTN manipulation rates than the microscopic approach in SS.^6^,^13^ In the microscopic group, inadvertent laceration of CTN occurred in one patient while resecting scutum. Despite the preservation of CTN integrity except for one patient, postoperative dysgeusia, significantly less in Group-I as compared Group-II, occurred owing to excessive surgical manipulations in both groups. Dysgeusia was improved spontaneously in six months the latest without any medical treatment.

Postoperative pain and vertigo are the two substantial complaints affecting the patients’ level of comfort after postoperatively. Postoperative pain and dizziness were reported to be less in ESS relative to MSS.^6^,^13^,^17^ Nevertheless, we have found no significant difference between groups regarding postoperative dizziness (Table-II). According to our opinion, rather than that the type of surgical approach, postoperative dizziness depends on the trauma severity during fenestration of footplate, the perilymph leak owing to the large fenestration, and prosthesis being longer than necessary. In our study, an endaural incision to obtain sufficient exposure was required in 11 patients with narrow and curved EAC in the microscopic group whereas it was not required in any of the patients having narrow and curved EAC in the endoscopic group. In Group-II, a higher level of pain relative to Group-I may be owing to endaural incisions. Moreover, ESS offers more minimally invasive surgery as compared microscopic approach.

More than one attempt during prosthesis placement was required due to manipulation difficulty related to one-handed surgery in the Group-I whereas the reason for multiple attempts for the prosthesis insertion in the Group-II was insufficient exposure of footplate. Once the surgeon gains experience in working with the endoscope, it may become easier to insert the prosthesis. On the other hand, even if experience increases in MSS, the insufficient exposure related to microscopic view will remain to be a reason for the difficulty of the prosthesis insertion particularly in patients with narrow and curved EAC. As the experience in ESS increases gradually, it may yield easier insertion of the prosthesis relative to the microscopic approach.

Finally, according to an experimental animal study, authors speculated that sensorineural hearing loss might occur owing to the temperature increase in the middle ear space in the subject during ESS.^18^ However, we feel the use of LED light sources with low heat dissipation capacity for illumination in endoscopes reduces the risk of heat increase in the middle ear space. There were no cases reported in the literature related to this subject, and none of the patients undergoing ESS in our study has SNHL.

In conclusion, ESS yields comparable audiometry outcomes, fewer complications, and more minimally invasive surgery, relative to the microscopic approach. Particularly, in patients with narrow and curved EAC in whom microscopic approach is challenging, ESS should be considered as a treatment option.

### Authors Contribution

**SG, EK:** Conceived, designed and did statistical analysis & editing of manuscript

**SG:** Did data collection and manuscript writing

**SG, EK:** Did review and final approval of manuscript.
